# Oligomerization Mechanisms of an H-NS Family Protein, Pmr, Encoded on the Plasmid pCAR1 Provide a Molecular Basis for Functions of H-NS Family Members

**DOI:** 10.1371/journal.pone.0105656

**Published:** 2014-08-19

**Authors:** Chiho Suzuki, Kohei Kawazuma, Shoichiro Horita, Tohru Terada, Masaru Tanokura, Kazunori Okada, Hisakazu Yamane, Hideaki Nojiri

**Affiliations:** 1 Biotechnology Research Center, The University of Tokyo, Tokyo, Japan; 2 Department of Applied Biological Chemistry, Graduate School of Agricultural and Life Sciences, The University of Tokyo, Tokyo, Japan; 3 Agricultural Bioinformatics Research Unit, Graduate School of Agricultural and Life Sciences, The University of Tokyo, Tokyo, Japan; INRA, France

## Abstract

Enterobacterial H-NS-like proteins and *Pseudomonas* MvaT-like proteins share low homology at the amino acid sequence level, but both can function as xenogeneic silencers and are included in the H-NS family of proteins. H-NS family members have dimerization/oligomerization and DNA-binding domains connected by a flexible linker and form large nucleoprotein complexes using both domains. Pmr, an MvaT-like protein encoded on the IncP-7 carbazole-degradative plasmid pCAR1, is a key regulator of an interaction between pCAR1 and its host *Pseudomonas putida* KT2440. KT2440 has two transcribed genes that encode the MvaT-like proteins TurA and TurB. Our previous transcriptome analyses suggested that the functions of Pmr, TurA and TurB are non-equivalent, although the detailed underlying mechanisms remain unclear. In this study, we focused on the protein–protein interactions of Pmr, and assessed the homo-oligomerization capacity of various substituted and truncated Pmr derivatives by protein–protein cross-linking analysis. Six of the seven residues identified as important for homo-oligomerization in Pmr were located near the N-terminus, and the putative flexible linker or the region near that was not involved in homo-oligomerization, suggesting that Pmr homo-oligomerization is different from that of enterobacterial H-NS and that the functional mechanism differs between H-NS-like and MvaT-like proteins. In addition, we assessed homo- and hetero-oligomerization of Pmr by surface plasmon resonance analysis and found that the coupling ratio of TurB-Pmr oligomers is smaller than that of Pmr-Pmr or TurA-Pmr oligomers. These results raised the possibility that composition of the hetero-oligomers of Pmr, TurA, and TurB could explain why the different gene sets were affected by either *pmr*, *turA*, or *turB* disruption in our previous studies.

## Introduction

Horizontal gene transfer (HGT) plays an important role in bacterial evolution and adaptation, generating tremendous bacterial diversity in nature. Because genes acquired through HGT may cause increased fitness costs, bacteria sometimes repress or “domesticate” the horizontally acquired genes [1]. H-NS, one of the nucleoid-associated proteins of bacteria, can repress the genes acquired through HGT by recognizing sequences with low GC content [2], [3]. In fact, genome-wide analyses have revealed a strong correlation between AT content and the binding sites of H-NS in *Escherichia coli* and *Salmonella enterica* serovar Typhimurium genomes [4]–[7]. Structurally, H-NS has two domains connected by a flexible linker: a dimerization/oligomerization domain at the N-terminal region and a DNA-binding domain at the C-terminal region [8]. When an H-NS dimer or oligomer binds to a specific high-affinity site using its C-terminal region, free soluble H-NS dimers or oligomers will bind the adjacent sites depending on the affinity of both the protein–protein interaction (N-terminal region of DNA-bound H-NS and that of free soluble H-NS) and protein-DNA interaction (C-terminal region of free soluble H-NS and DNA) [9]–[11]. The resulting H-NS nucleoprotein complexes are considered to mediate gene silencing by preventing open complex formation by RNA polymerase or trapping the open complex once it has formed [12], [13].

H-NS is also encoded on some plasmids, particularly those with low GC content [14]. Recent studies have shown that plasmid-encoded nucleoid-associated proteins, including H-NS, play important roles in transcriptional regulation networks between plasmids and host chromosomes and in maintaining host cell fitness [15]. For example, Sfh, an H-NS-like protein encoded on the conjugative IncHI1 plasmid pSfR27, can function as a stealth protein that allows the transfer of pSfR27 without causing a major effect on gene expression in a recipient [16]. Genome-wide chromatin immunoprecipitation coupled with microarray (ChIP-chip) analysis showed that all of the DNA binding sites of Sfh were completely overlapped with those of H-NS [17]. However, in the absence of H-NS, Sfh interacts with a wider population of H-NS targets, suggesting that Sfh may play a molecular “backup” role for H-NS [17]. H-NS-like protein encoded on plasmid R27 (H-NS_R27_) selectively targets HGT DNA and not core genome DNA, whereas chromosomally encoded H-NS targets both [18]. Note that plasmid R27 is 99.7% identical to pSfR27, and H-NS_R27_ is 98% identical to Sfh.

MvaT-like proteins of pseudomonads have low amino acid sequence homology with H-NS (<25% identity) but can complement an *hns*-related phenotype of *E. coli* [19]. This explains why these proteins are members of “H-NS family proteins”, which include both H-NS-like proteins and its functional homologs [3], [19], [20], although it remains unknown whether the mechanisms for their function are the same among H-NS family proteins. In *Pseudomonas aeruginosa* PAO1, MvaT and its homologous protein MvaU were shown to interact with each other, bind to the same chromosomal regions, and function coordinately [21], [22]. This strain cannot tolerate the loss of both MvaT and MvaU because it results in the induction of Pf4 phage, which superinfects and kills cells or inhibits their growth [23]. Conversely, *P. putida* KT2440 has five genes encoding MvaT-like proteins, *turA-E*, although transcriptional levels of *turC*, *turD*, and *turE* were considerably lower than those of *turA* and *turB* [24]–[27].

**Figure 1:**
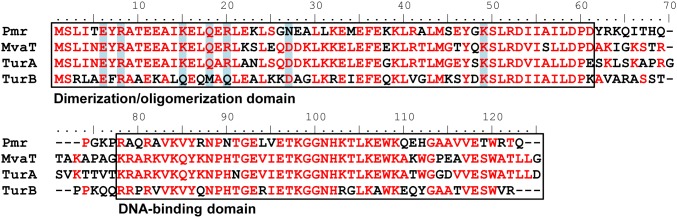
Alignments of amino acid sequences of Pmr, TurA, TurB, and MvaT of *P. aeruginosa* PAO1. Alignments were performed using the CLUSTAL W software, version 2.1. The amino acid residues identical in two to four members are shown in red. Dimerization/oligomerization and DNA-binding domains of Pmr and the corresponding regions in the other three proteins are indicated in the panel. The seven residues important for homo-oligomerization of Pmr, and the corresponding residues in the other three proteins, are highlighted in light blue.

The completely sequenced IncP-7 carbazole-degradative plasmid pCAR1, which was isolated originally from *P. resinovorans* CA10, carries a gene encoding an MvaT-like protein, Pmr, which shows 58% identity with MvaT of *P. aeruginosa* PAO1 and is a key regulator of pCAR1 and its host *Pseudomonas* functions [28]. In a previous study, we used *P. putida* KT2440 as a model host strain and found that Pmr preferentially binds to HGT DNA and affects gene transcription both on the plasmid and host chromosome [27]. We also found that Pmr forms homo-oligomers consisting of its homodimers and hetero-oligomers with TurA and TurB *in vitro* [27], [29]. Interestingly, although Pmr, TurA, and TurB have high amino acid sequence homology (58% identity between Pmr and TurA, 53% identity between Pmr and TurB, and 50% identity between TurA and TurB; Figure 1), each of these three MvaT-like proteins has unique regulons, suggesting that their functions are non-equivalent (Yun *et al.*, unpublished data). To clarify the molecular mechanisms underlying the functions of the three MvaT-like proteins, it is necessary to evaluate and compare their protein–protein and protein–DNA affinities.

In the present study, we focused on the protein–protein interactions of Pmr. To clarify Pmr homo-oligomerization, the homo-oligomerization capacity of various substituted and truncated derivatives of Pmr was assessed. The distribution of the residues that are important for homo-oligomerization of Pmr was different from not only that of enterobacterial H-NS but also that of MvaT of *P. aeruginosa* PAO1, suggesting that Pmr has a novel oligomerization mechanism in H-NS family proteins. In addition, to gain insight into the hetero-oligomerization mechanism of Pmr, we determined coupling ratios of Pmr-Pmr, TurA-Pmr, and TurB-Pmr combinations using the surface plasmon resonance (SPR) analysis, and found that the coupling ratios of Pmr-Pmr or TurA-Pmr oligomers and TurB-Pmr oligomers are different. This result raised the possibility that composition of the hetero-oligomers of Pmr, TurA, and TurB could explain why the different gene sets were affected by either *pmr*, *turA*, or *turB* disruption in our previous studies.

## Results

### Identification of the dimerization/oligomerization domain of Pmr

**Figure 2:**
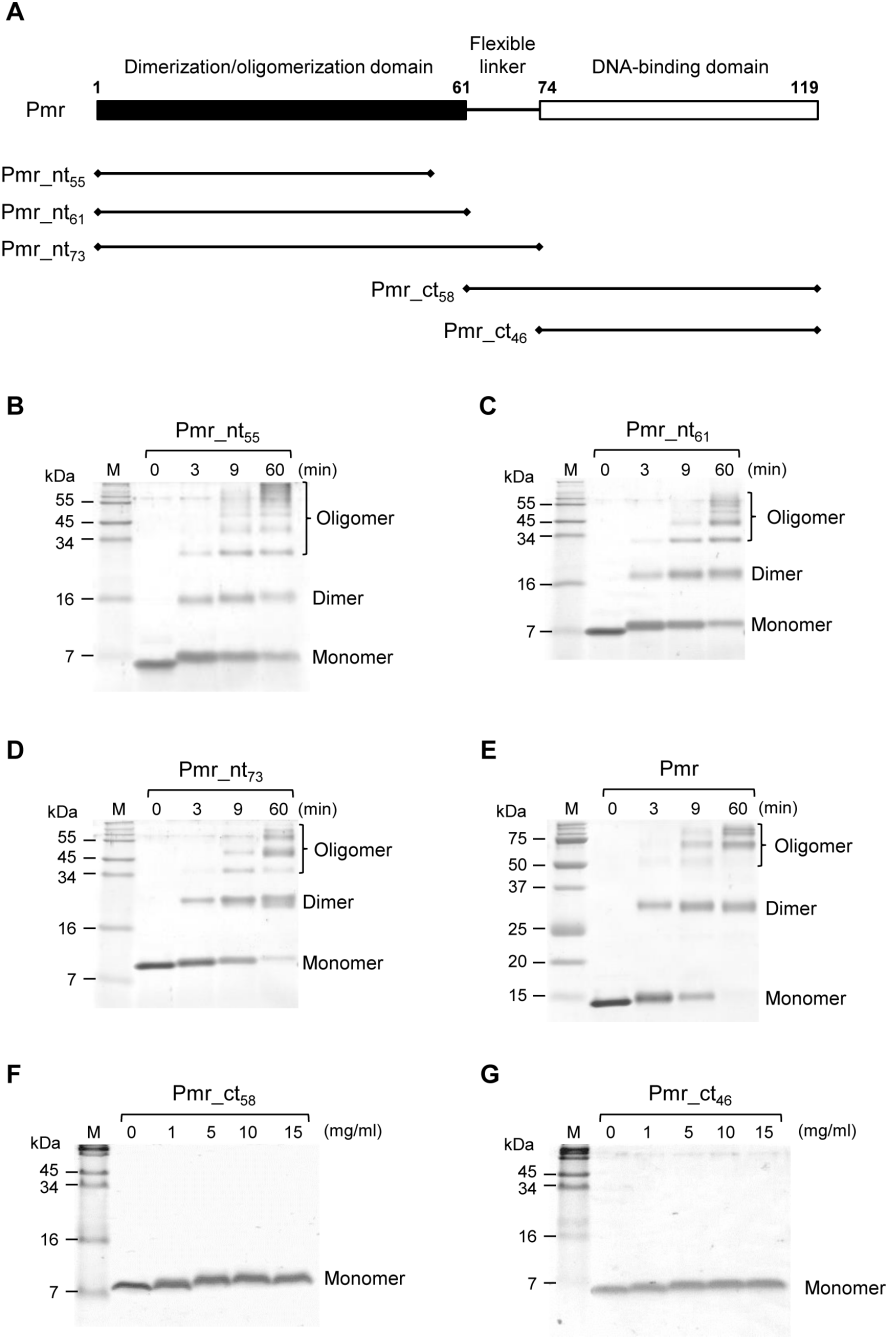
Effect of truncation on the homo-oligomerization capacity of Pmr. (A) Schematic representation of Pmr derivatives. Putative domain organization and a set of C-terminally or N-terminally truncated forms of Pmr, characterized in this study, are shown. Numbers indicate positions of amino acids. (B)–(G) Tricine-SDS-PAGE profiles of Pmr_nt_55_ (B), Pmr_nt_61_ (C), Pmr_nt_73_ (D), Pmr (E), Pmr_ct_58_ (F), and Pmr_ct_46_ (G) after chemical cross-linking. The analyses were performed using DMS as a cross-linker. Numbers indicate incubation durations (min) with DMS (B–E) or concentrations (mg/ml) of DMS (F, G). “M” indicates the protein marker. The bands corresponding to oligomers, dimers, and monomers are indicated.

To identify the dimerization/oligomerization domain of Pmr, we compared homo-oligomerization capacity of truncated Pmr derivatives. Alignment of the amino acid sequences of Pmr, TurA, TurB, and MvaT of *P. aeruginosa* PAO1 revealed that residues 1–61 and 74–119 were highly conserved, whereas residues 62–73 of Pmr were less conserved (Figure 1). Since H-NS has N-terminal dimerization/oligomerization domain and C-terminal DNA-binding domain connected by a flexible linker [8], we expected that residues 1–61, 62–73, and 74–119 of Pmr correspond to dimerization/oligomerization domain, flexible linker, and DNA-binding domain, respectively. In fact, the C-terminal region of Pmr (residues 74–119) exhibited DNA-binding activity in our previous study [29]. To confirm that the N-terminal region of Pmr contains dimerization/oligomerization domain, we prepared heterologously expressed Pmr derivatives which have residues 1–61 (Pmr_nt_61_, putative dimerization/oligomerization domain), 1–73 (Pmr_nt_73_, putative dimerization/oligomerization domain and putative flexible linker), 62–119 (Pmr_ct_58_, putative flexible linker and DNA-binding domain), and 74–119 (Pmr_ct_46_, DNA-binding domain) (Figure 2A). We also prepared a Pmr derivative which has residues 1–55 (Pmr_nt_55_), because a previous report showed that the first 55 residues of MvaT are not sufficient for homo-oligomerization [30]. These proteins were purified by metal chelate affinity chromatography, and their homo-oligomerization capacity was assessed by protein–protein chemical cross-linking using dimethyl suberimidate dihydrochloride (DMS) followed by Tricine-SDS-PAGE. Similar to the full-length Pmr, more cross-linked homodimers and homo-oligomers of Pmr_nt_55_, Pmr_nt_61_, and Pmr_nt_73_ were detected, depending on the time of incubation (Figure 2B–E). Nevertheless, the observed differences in cross-linking efficiency could be attributed to the increasing number of lysine residues (6, 6, 8, and 13 in Pmr_nt_55_, Pmr_nt_61_, Pmr_nt_73_, and Pmr, respectively), which are targets of DMS. On the other hand, no cross-linked homodimer or homo-oligomer was detected when Pmr_ct_58_ or Pmr_ct_46_ was analyzed (Figure 2F and G). We note that the apparent molecular mass of the proteins, particularly that of monomers, increased depending on the incubation time and the concentration of DMS, probably due to DMS binding to (but not cross-linking between) the proteins. These results indicated that residues 62–119 are dispensable for homo-oligomerization. Considering that residues 1–61 were highly conserved with other MvaT homologs (Figure 1), we identified Pmr_nt_61_ as the dimerization/oligomerization domain of Pmr.

### Identification of amino acid residues responsible for homo-oligomerization of Pmr_nt_61_

**Figure 3:**
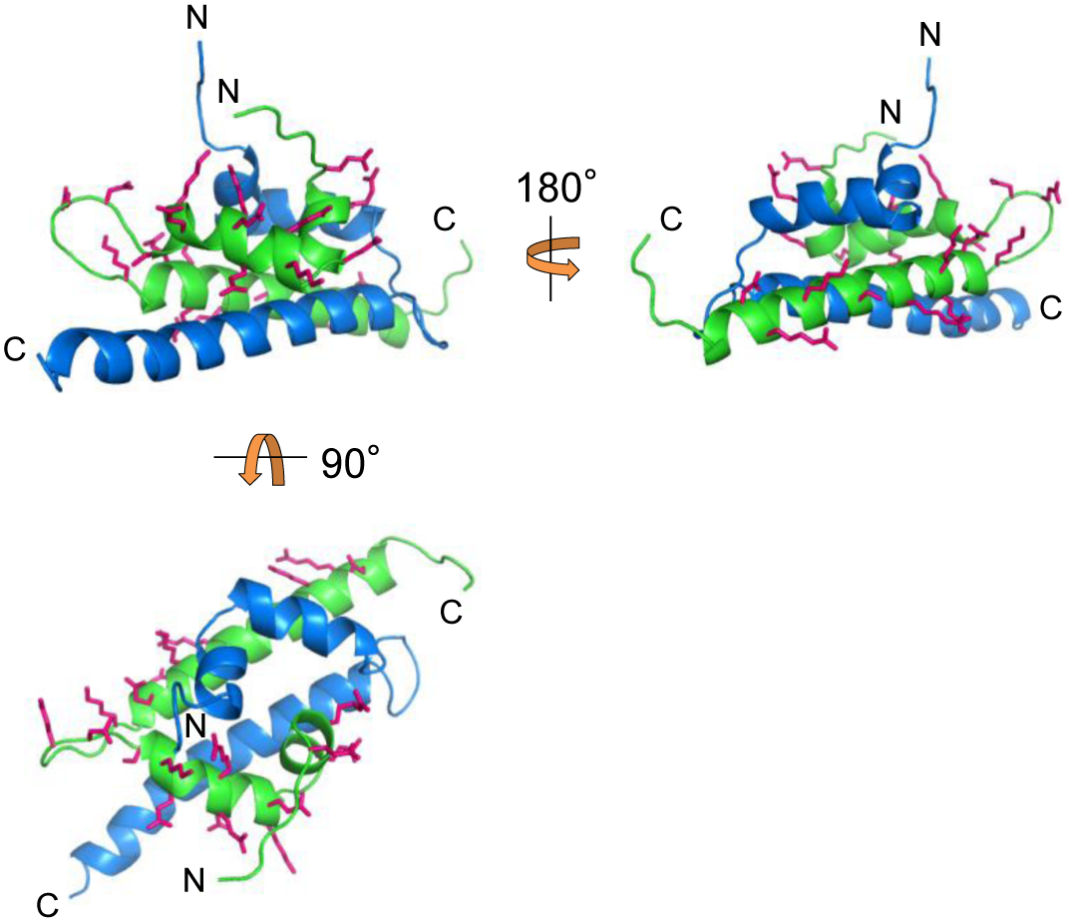
Ribbon representation of the structural model of the Pmr_nt_61_ dimer. The two protomers are displayed in green and blue. On the green protomer, side chains of the 22 residues predicted to be important for homo-oligomerization are shown in magenta. The N- and C-terminus of the two protomers are shown as “N” and “C”, respectively.

**Figure 4:**
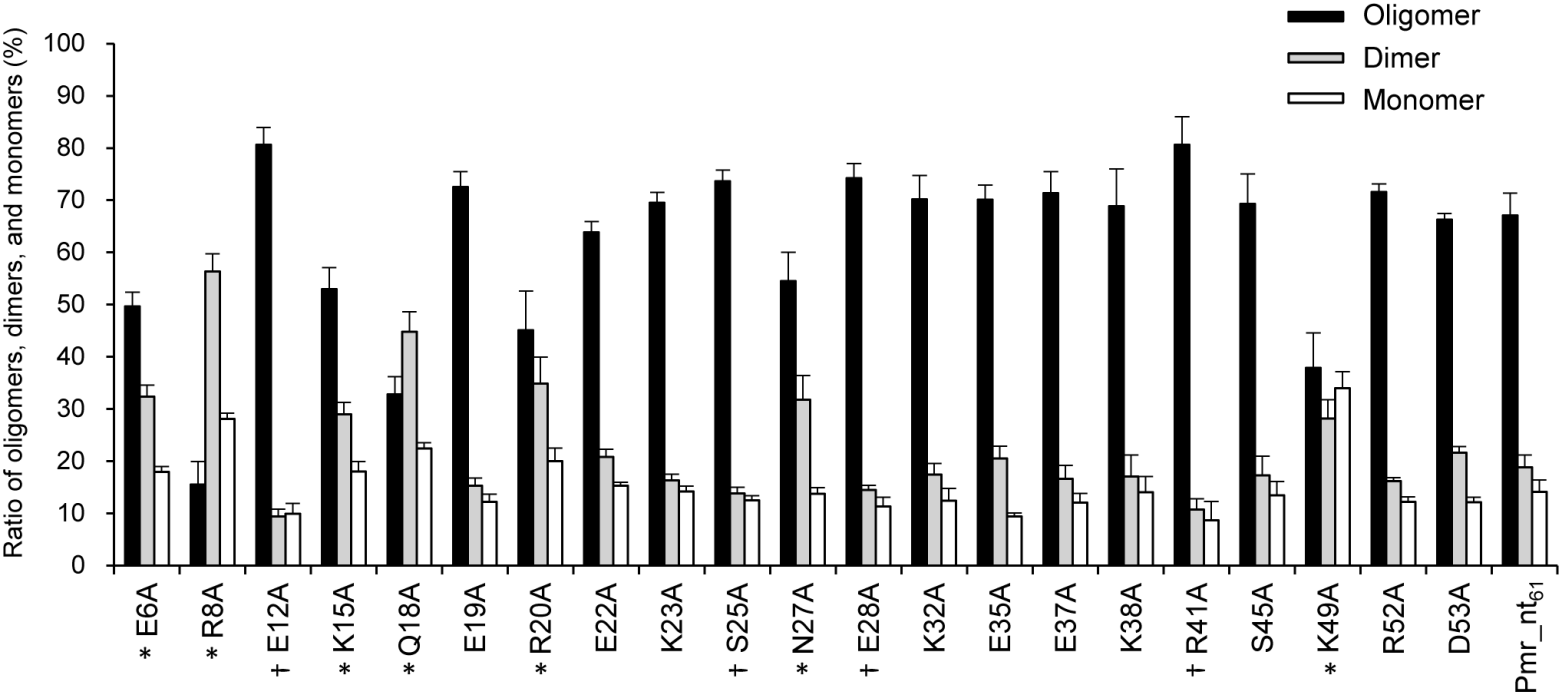
Ratio of oligomers, dimers, and monomers of Pmr_nt_61_ and its variants. After protein–protein cross-linking, samples were analyzed by Tricine-SDS-PAGE, and the intensity of the bands in the 60-min lane was quantified using the ImageJ software (http://rsbweb.nih.gov/ij/). Pmr_nt_61_ variants are indicated, such as “E6A” for Pmr_nt_61_-E6A. Data are expressed as means ± standard deviation from three (for Pmr_nt_61_ variants) or five (for native Pmr_nt_61_) independent experiments. Black, gray, and white bars show the percentages of oligomers, dimers, and monomers, respectively. * and † indicate Pmr_nt_61_ variants that had significant decreases and increases, respectively, in the ratio of oligomers compared with native Pmr_nt_61_ (P<0.05, two-tailed Student’s *t*-test).

We next identified the amino acid residues responsible for homo-oligomerization of Pmr_nt_61_. Here, we first constructed the structural model of the dimer form of Pmr_nt_61_ as described in the “Materials and Methods” section using the structure of an H-NS-like protein of *Vibrio cholerae*, VicH [31], as a template (Figure 3). In the structural model, most of the side chains of hydrophobic residues constituted the dimer-forming surface, such as that in VicH. Hence, we expected that the charged residues on the surface of the dimer model would be important for homo-oligomerization of Pmr_nt_61_. We focused on the 22 charged residues among the 61 total residues, the side chains of which are predicted to be exposed to the solvent (Figure 3). To determine whether these residues were important for homo-oligomerization of Pmr_nt_61_, the following 22 single amino acid substitutions were introduced into Pmr_nt_61_: E6A, R8A, E12A, K15A, E16A, Q18A, E19A, R20A, E22A, K23A, S25A, N27A, E28A, K32A, E35A, E37A, K38A, R41A, S45A, K49A, R52A, and D53A. Protein–protein chemical cross-linking followed by Tricine-SDS-PAGE was performed on these variants, with the exception of Pmr_nt_61_-E16A, which was almost insoluble when expressed in *E. coli* (data not shown). In some of the variants, the ratio of oligomers, dimers, and monomers changed compared with native Pmr_nt_61_ (Figure S1). To quantify the ratio of oligomers, dimers, and monomers, the intensities of the Tricine-SDS-PAGE bands in the 60-min lane were quantified using the ImageJ software (http://rsbweb.nih.gov/ij/) (Figure 4). Compared with the native protein (Pmr_nt_61_), the ratio of oligomers increased significantly in four variants (Pmr_nt_61_-E12A, -S25A, -E28A, and -R41A), and decreased significantly in seven variants (Pmr_nt_61_-E6A, -R8A, -K15A, -Q18A, -R20A, -N27A, and -K49A) (P<0.05, two-tailed Student’s *t*-test). Regarding the latter variants, the ratio of all the dimers and that of the monomers of six variants (except for Pmr_nt_61_-N27A) increased significantly (P<0.05, two-tailed Student’s *t*-test). Therefore, we concluded that the seven Pmr_nt_61_ variants the oligomer ratios of which decreased significantly were oligomerization-deficient proteins, and the corresponding seven residues, E6, R8, K15, Q18, R20, N27, and K49, were demonstrated to be important for homo-oligomerization of Pmr_nt_61_. Although the reason that the ratio of oligomers of the other four Pmr_nt_61_ variants increased was unclear, we focused on the seven oligomerization-deficient proteins and performed the following experiments.

### The putative flexible linker of Pmr was not involved in homo-oligomerization

**Figure 5:**
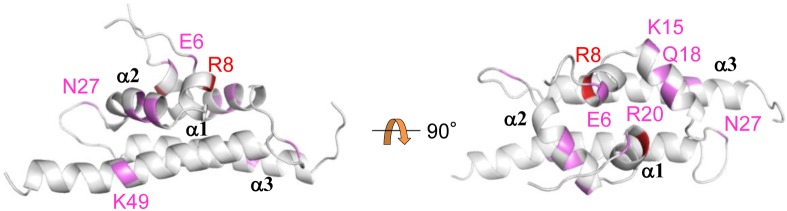
Distribution of the seven amino acid residues important for homo-oligomerization of Pmr_nt_61_. The seven residues are highlighted in the structural model of the Pmr_nt_61_ dimer. R8 is shown in red because its effect on the homo-oligomerization of Pmr_nt_61_ was the greatest among the seven residues. The other residues are shown in pink. “α1”, “α2”, and “α3” are the names of helices from the N-terminus of a Pmr_nt_61_ protomer.

Among the seven residues that were important for homo-oligomerization of Pmr_nt_61_, six residues, the exception being K49, were located in the α1 and α2 helices, as well as the adjacent loops, in the structural model (Figure 5). This result indicates that Pmr oligomerizes mainly using the two helices in the N-terminal region. Conversely, the crystal structure of the N-terminal region of H-NS suggested that H-NS oligomerizes using the two dimerization sites: N-terminal helices H1 and H2 (referred to as “Site 1”) and helices H3 and H4 near the flexible linker (referred to as “Site 2”) (Figure S2A) [32]. Because Site 2 of H-NS was previously recognized as a part of the flexible linker [33], we could not simply argue that the region near the putative flexible linker (or itself) of Pmr was not concerned with homo-oligomerization. Since Pmr_nt_61_ (dimerization/oligomerization domain without the putative flexible linker) formed homo-oligomers and Pmr_ct_58_ (DNA-binding domain with the putative flexible linker) did not form homo-oligomers (Figure 2), we focused on the subordinate contribution of the putative flexible linker for the homo-oligomerization.

**Figure 6:**
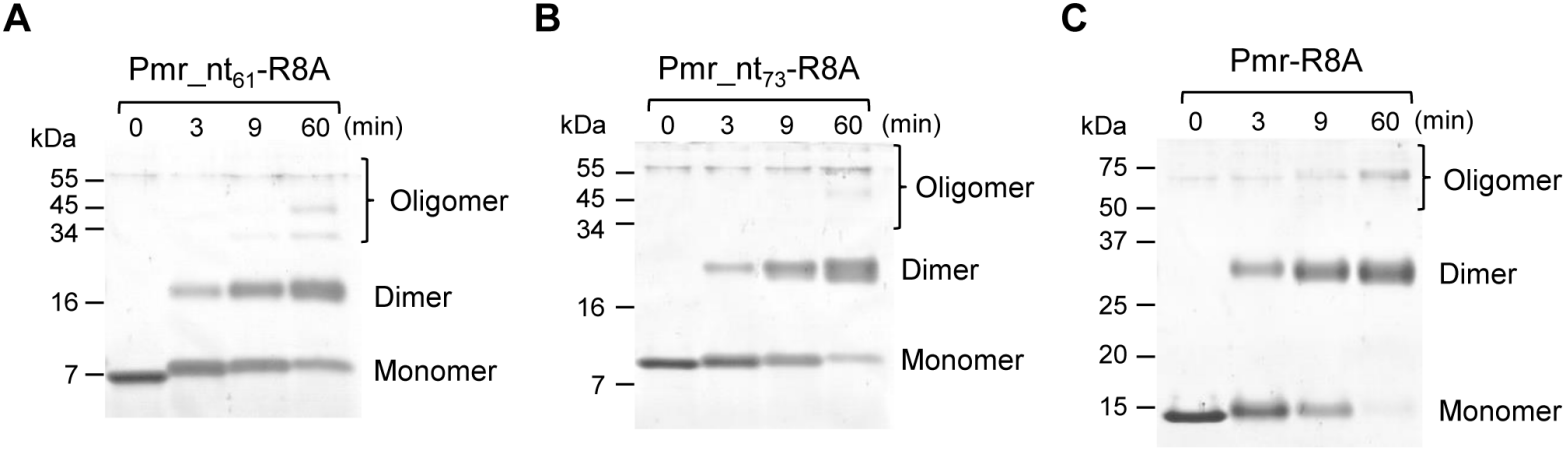
Effect of amino acid substitution on the homo-oligomerization capacity of Pmr_nt_61_, Pmr_nt_73_, and Pmr. Tricine-SDS-PAGE profiles of Pmr_nt_61_-R8A (A), Pmr_nt_73_-R8A (B), and Pmr-R8A (C) after chemical cross-linking are shown. The analyses were performed using DMS as a cross-linker. Numbers indicate incubation durations (min).

**Figure 7:**
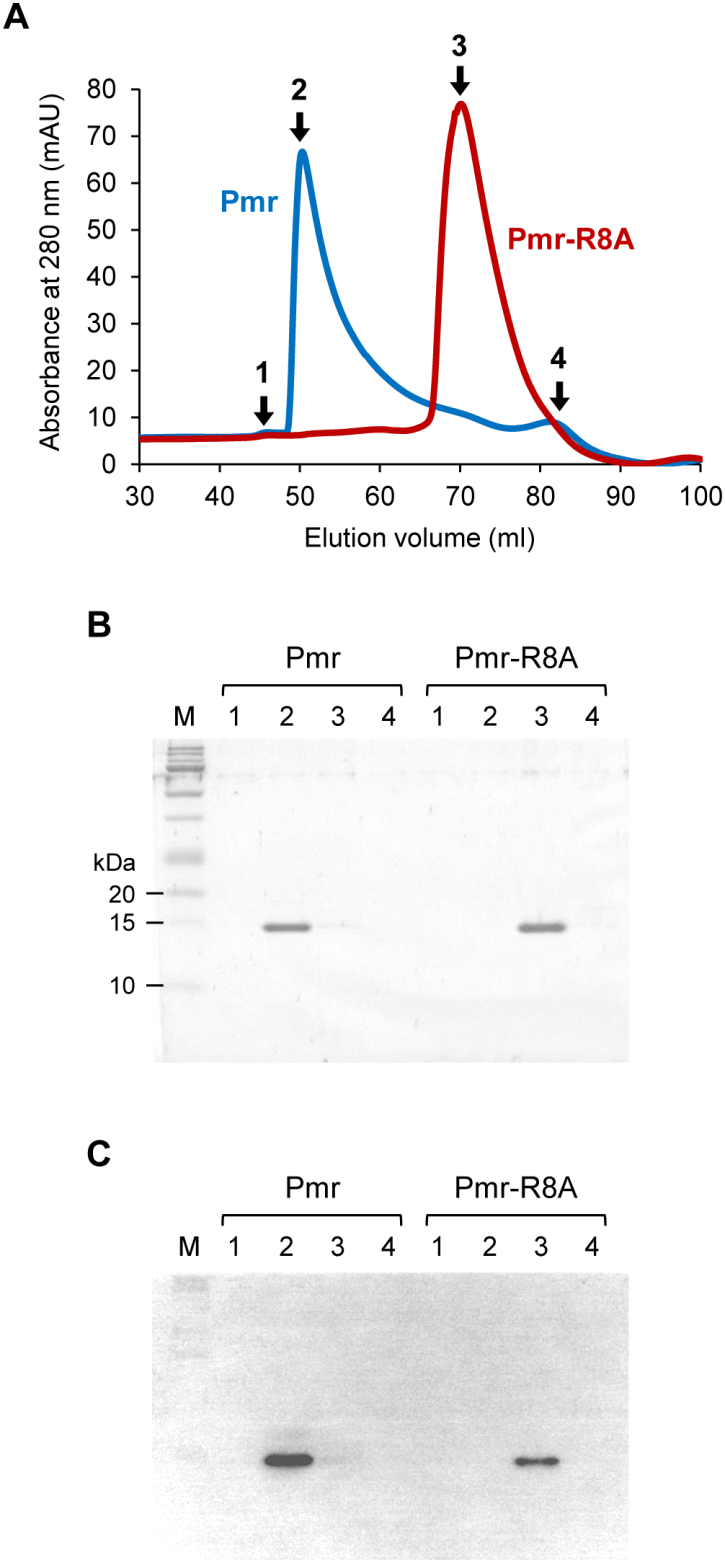
Gel-filtration chromatography of Pmr and Pmr-R8A. (A) Elution profiles of Pmr (blue line) and Pmr-R8A (red line). The figures present the absorbance at 280 nm as a function of the elution volume (ml). One milliliter of purified proteins at 140 µM was applied to a HiLoad 16/60 Superdex 200 prep-grade column in buffer B (20 mM Tris-HCl (pH 7.5, 4°C), 0.5 M NaCl, 10% glycerol, and 0.5 M imidazole). The numbers indicate the fractions which were applied to Tricine-SDS-PAGE and Western blot analysis. (B) Tricine-SDS-PAGE profiles of fractions from gel filtration chromatography. The numbers correspond to elution profiles shown in panel A. Ten microliters were applied to Tricine-SDS-PAGE from 1 ml of the fraction. “M” indicates the protein marker. (C) Western blot analysis using anti-His antibody and the same samples in Tricine-SDS-PAGE shown in panel B.

We compared the oligomerization capacity of Pmr_nt_61_-R8A and that of Pmr_nt_73_-R8A (dimerization/oligomerization domain with the putative flexible linker) and Pmr-R8A. Pmr_nt_73_-R8A and Pmr-R8A harbor the same substitution as Pmr_nt_61_-R8A, which formed the smallest amount of homo-oligomers in the amino acid residue replacement studies (Figure 4). If the putative flexible linker had subordinate function for homo-oligomerization, Pmr_nt_73_-R8A and Pmr-R8A would form more oligomers than Pmr_nt_61_-R8A did. However, Pmr_nt_73_-R8A and Pmr-R8A formed only a small amount of oligomers like Pmr_nt_61_-R8A did (Figure 6), suggesting that the putative flexible linker was not involved in Pmr homo-oligomerization. To confirm that the above experiments evaluated not the efficiency of cross-linking but the homo-oligomerization capacity of the proteins, we also performed gel-filtration chromatography using Pmr and Pmr-R8A (Figure 7). Native Pmr formed oligomeric species corresponding to a ∼35-mer (520 kDa, shown as “2” in Figure 7), whereas Pmr-R8A formed oligomeric species corresponding to a ∼6-mer (94 kDa, shown as “3” in Figure 7). Taken together, the results showed that the region important for Pmr homo-oligomerization was mainly distributed in the N-terminal helices α1 and α2, and the putative flexible linker was not involved in homo-oligomerization.

### Coupling ratios of Pmr-Pmr and TurA-Pmr oligomers differed from that of TurB-Pmr oligomers

To gain insight into the hetero-oligomerization mechanism of Pmr, we assessed whether the seven amino acid residues important for homo-oligomerization of Pmr_nt_61_ were conserved in TurA or TurB. As shown in Figure 1, although six of the seven residues were conserved in TurA, only three of the seven were conserved in TurB, suggesting that the homo-oligomerization function of TurA was similar to that of Pmr, whereas that of TurB was somewhat different. If Pmr and TurA have similar homo-oligomerization mechanisms, and the residues important for hetero-oligomerization are the same as those for homo-oligomerization, they will form hetero-oligomers using mechanisms similar to those involved in formation of homo-oligomers. Conversely, if TurB has a different homo-oligomerization mechanism than Pmr or TurA, Pmr and TurB will form hetero-oligomers using different mechanisms. Hence, we determined the coupling ratios of Pmr-Pmr, TurA-Pmr, and TurB-Pmr combinations using the SPR technique.

**Figure 8:**
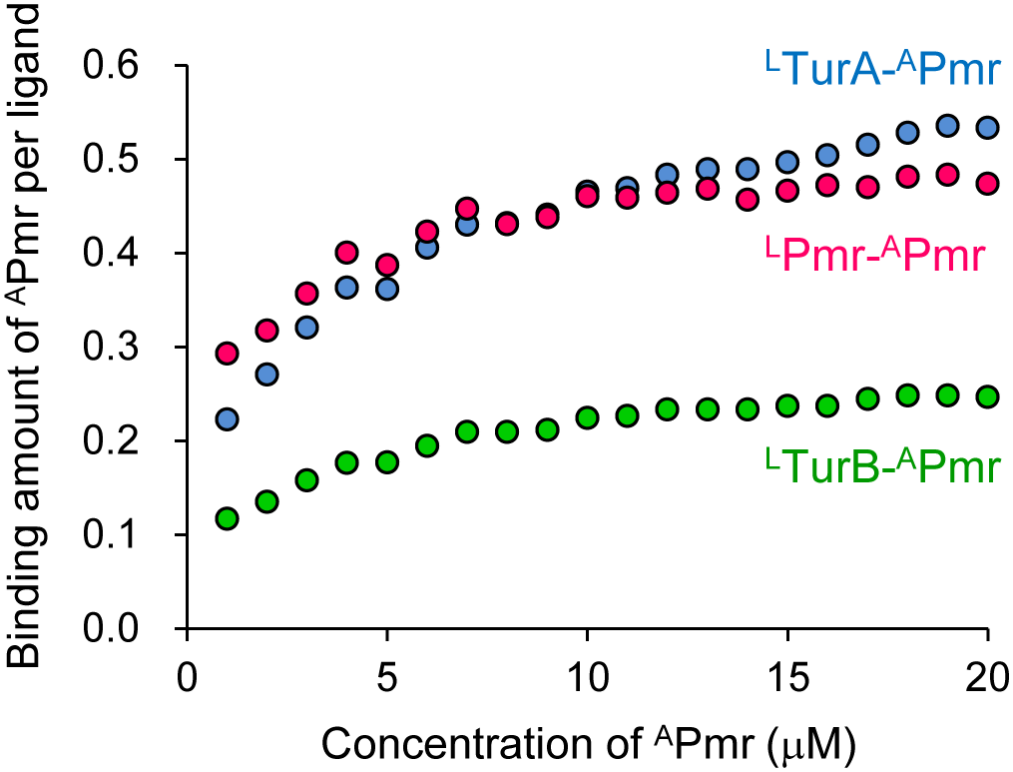
Coupling ratios of Pmr-Pmr, TurA-Pmr, and TurB-Pmr oligomers. The figure presents the binding amount of ^A^Pmr per one homodimer of ^L^Pmr (pink), ^L^TurA (blue), and ^L^TurB (green) as a function of the concentration of ^A^Pmr (µM). The immobilized amount of ^L^Pmr, ^L^TurA, and ^L^TurB were 530 RU, 540 RU, and 540 RU, respectively. To calculate the binding amount of ^A^Pmr per one homodimer of the ligands, the equilibrium binding response (*R*_eq_) was divided by the amount of immobilized ^L^Pmr, ^L^TurA, and ^L^TurB.

To analyze the interaction, biotinylated Pmr, TurA, and TurB (as ligands, indicated as ^L^Pmr, ^L^TurA, and ^L^TurB) was immobilized separately in three flow cells of a streptavidin-coated sensor chip as described in the “Materials and Methods” section, and various Pmr concentrations (as analyte, indicated as ^A^Pmr) were injected into the sensor chip. The three MvaT-like proteins form homo-oligomers consisting of many homodimers, and the homodimers are expected to repeat association-dissociation processes in solution [29]. Therefore, the immobilized ^L^Pmr, ^L^TurA, and ^L^TurB are regarded to be homodimers; when one of the biotinylated homodimers in a large homo-oligomer binds to the surface of a sensor chip, the other homodimers will dissociate from the immobilized one in the continuous-flow microfluidics. On the other hand, the oligomerization state of ^A^Pmr is not uniform. Hence, we immobilized nearly identical amounts of ^L^Pmr, ^L^TurA, and ^L^TurB (about 500 resonance units, RU) separately in the three flow cells, and evaluated the binding amount of ^A^Pmr using the same ^A^Pmr solutions in the three flow cells simultaneously. When ^A^Pmr was applied to the sensor chip, binding equilibrium was achieved during the injection (data not shown). To calculate the binding amount of ^A^Pmr per one homodimer of ^L^Pmr, ^L^TurA, and ^L^TurB, the equilibrium binding response (*R*_eq_) was divided by the amount of immobilized ^L^Pmr, ^L^TurA, and ^L^TurB (i.e. about 500 RU). When higher ^A^Pmr concentrations were used, the binding amount of ^A^Pmr kept increasing, probably because of homo-oligomerization of ^A^Pmr itself (Figure S3). To obtain data which reflect ligand-analyte interaction, we used lower ^A^Pmr concentrations to calculate the binding amount of ^A^Pmr (Figure 8). When the ligand-analyte interaction reached equilibrium, the binding amount of ^A^Pmr in ^L^Pmr-^A^Pmr and ^L^TurA-^A^Pmr combinations were twice that in ^L^TurB-^A^Pmr, and there was no significant difference between the values in the ^L^Pmr-^A^Pmr and ^L^TurA-^A^Pmr combinations (Figure 8). These results suggest that the coupling ratios of Pmr-Pmr or TurA-Pmr oligomers and TurB-Pmr oligomers are different.

## Discussion

### Pmr has a novel oligomerization mechanism in H-NS family proteins

High-order oligomerization is one of the important features of the gene-silencing function of H-NS family proteins. In fact, H-NS or MvaT variants, which lack the ability to form higher-order oligomers, were defective for gene silencing [30], [34]. Both domains, which are responsible for higher-order oligomer formation and dimer formation, have been shown to exist in the N-terminal region of H-NS and MvaT [30], [32], [34]–[36]. In the present study, we found that Pmr also has a dimerization/oligomerization domain in its N-terminal region, similar to H-NS and MvaT (Figure 2). To elucidate the homo-oligomerization mechanism of Pmr, we generated 22 Pmr_nt_61_ variants and evaluated their homo-oligomerization capacity using protein–protein cross-linking (Figure 4). Seven of the 22 residues were identified as important for homo-oligomerization of Pmr_nt_61_. Because six of the seven residues were located in helices α1 and α2, as well as the adjacent loops of the structural model of Pmr_nt_61_, Pmr was expected to oligomerize using mainly its N-terminal helices. Recently, Arold *et al.* solved the crystal structure of the N-terminal region of H-NS of *S. enterica* Typhimurium in an oligomerized state and found that it forms a superhelical structure [32]. In this structure, H-NS oligomerizes using N-terminal helices H1 and H2 (Site 1, residues 3–19) and the C-terminal part of the helix H3 and helix H4 (Site 2, residues 67–83), as described in the “Results” section (Figure S2A) [32]. When either Site 1 or Site 2 was truncated, H-NS formed only homodimers and could not form homo-oligomers [35], [36]. If the homo-oligomerization mechanism of Pmr is similar to that of H-NS, Pmr should have both Sites 1 and 2. Because six of the seven residues that are important for homo-oligomerization of Pmr were found to be located in helices α1 and α2, as well as the adjacent loops in the structural model (Figure 5), these regions are expected to be Site 1 of Pmr. Conversely, Site 2 of Pmr was expected to be included in helix α3 (residues 33–61) or in the putative flexible linker (residues 62–73). Because the existence of the putative flexible linker did not affect the homo-oligomerization capacity of substituted and truncated Pmr derivatives (Figure 2D, 2F, and 6), the putative flexible linker of Pmr was found to be dispensable for homo-oligomerization. Since Pmr_nt_55_ formed homo-oligomers (Figure 2B), residues 56–61 (the C-terminal part of helix α3) are also dispensable for homo-oligomerization. Among the residues 33–55, only one substitution (K49A) from the eight substitutions decreased homo-oligomerization capacity of Pmr_nt_61_ (Figure 4). Although we could not exclude the possibility that the other residues in helix α3 are important for homo-oligomerization of Pmr_nt_61_, it is highly likely that Pmr does not have Site 2. These results suggest that the homo-oligomerization mechanism of Pmr is different from that of H-NS in *E. coli* or *S. enterica* Typhimurium.

Additionally, the distribution of the residues important for homo-oligomerization of Pmr was different from that of MvaT of *P. aeruginosa*. Regarding MvaT, the region spanning residues 2–35 is involved in dimer formation, and the region spanning residues 35–62 mediates higher-order oligomer formation, suggesting that the homo-oligomerization mechanism of MvaT is similar to that of H-NS [30]. In addition, the bacterial two-hybrid assay showed that the first 55 residues of MvaT were not sufficient for homo-oligomerization [30], whereas Pmr_nt_55_ could form homo-oligomers (Figure 2B). In MvaT, six of the seven residues important for homo-oligomerization of Pmr were conserved, and the dimerization/oligomerization domain has 70% amino acid sequence identity with Pmr (Figure 1). Although the difference between MvaT and Pmr could be caused by the difference in experimental methods or conditions, some residues that are not conserved between the two proteins might lead to different homo-oligomerization mechanisms. These results raised the possibility that, although H-NS family proteins have functional similarity, the molecular mechanisms of their functions are different. Unfortunately, at present no structural information for MvaT-like proteins is available, and so it is difficult to discuss the difference between the homo-oligomerization mechanisms of H-NS and MvaT or between MvaT and Pmr. Structural information is required to clarify the mechanisms of Pmr homo-oligomerization.

### Different coupling ratios might explain the functional differences among the three MvaT-like proteins

Many bacteria have more than one H-NS family protein. Additionally, in some cases, they also have a plasmid that encodes an H-NS family protein. These additional H-NS family proteins are regarded as molecular “backup” [16], [17], [37], [38]. Conversely, some evidence indicates that the functions of the multiple H-NS family proteins are distinct [18], [39]–[41]. Similarly, pCAR1-encoded Pmr and chromosomally encoded TurA and TurB have unique regulons, suggesting that functions of these three MvaT-like proteins are non-equivalent (Yun *et al.*, unpublished data). However, the binding sites of Pmr, TurA, and TurB detected by chromatin affinity purification coupled with high-density tiling chip (ChAP-chip) analyses were almost identical, and we could not explain the functional differences among the three MvaT-like proteins by their *in vivo* DNA binding sites (Yun *et al.*, unpublished data) [27]. In the present study, we performed SPR studies to gain insight into the hetero-oligomerization mechanisms of Pmr. When Pmr, TurA, and TurB was immobilized on a sensor chip and various Pmr concentrations were injected, coupling ratios of Pmr-Pmr or TurA-Pmr oligomers and TurB-Pmr oligomers were suggested to be different (Figure 8). In *P. putida* KT2440(pCAR1) cells, Pmr, TurA, and TurB dimers are thought to bind to a specific high-affinity site (motivated by protein-DNA interaction), and then spread along DNA by the binding of additional Pmr, TurA, and TurB dimers (motivated by both protein-DNA and protein–protein interactions). These findings suggest that when free Pmr dimers are approaching DNA-bound Pmr, TurA, or TurB, the composition of the resultant hetero-oligomers will differ among the following three combinations: Pmr or TurA will form oligomers with twice as many Pmr molecules as those of TurB. When TurA or TurB dimers approach DNA-bound MvaT-like proteins, they will form oligomers depending on the coupling ratio of each combination. In addition, the affinity of the protein–DNA interaction also affects the composition of the hetero-oligomers, although the effect will be minor if the high-affinity DNA sequences of the three MvaT-like proteins are similar. The resultant various hetero-oligomers are detected as the binding sites of Pmr, TurA, and TurB in ChAP-chip analyses, although their composition differed. However, in *pmr-*, *turA-*, or *turB-*disrupted mutants of *P. putida* KT2440(pCAR1), the composition of the hetero-oligomers will be altered. Such a change in the composition of each protein in the hetero-oligomers may lead to an alteration in the region covered by the three MvaT-like proteins. We consider that this may explain why the different gene sets were affected by either *pmr*, *turA*, or *turB* disruption.

Our *in vitro* analysis shed light on the molecular mechanism underlying the functional differences among the three MvaT-like proteins. However, some questions remain to be clarified. The first concerns the molecular mechanism underlying the different coupling ratio of TurB-Pmr. As mentioned in the “Results” section, six of the seven amino acid residues that are important for homo-oligomerization of Pmr are conserved in TurA, whereas only three are conserved in TurB (Figure 1). In terms of the distributions of these residues, K15, Q18, and R20, which are located on helix α2 in the structural model (Figure 5), are not conserved in TurB (Figure 1). At first, we hypothesized that these residues to account for the difference between TurA and TurB, and so we generated the TurA^K15Q/Q18M/R20Q^ protein, in which K15, Q18, and R20 of TurA were substituted with the corresponding residues of TurB. However, there was no significant difference in the coupling ratio between ^L^TurA-^A^Pmr and ^L^TurA^K15Q/Q18M/R20Q^-^A^Pmr in SPR studies, although the homo-oligomerization capacity of TurA^K15Q/Q18M/R20Q^ was significantly lower than that of native TurA (data not shown). These results suggest that the amino acid residues important for homo-oligomerization of TurA are different from those for hetero-oligomerization of TurA and Pmr. To clarify the difference between TurA and TurB, it is necessary to assess their homo-oligomerization mechanisms, as well as the regions important for hetero-oligomerization in Pmr, TurA, and TurB. Another question is whether the recognition sequences of Pmr, TurA, or TurB dimers are identical. As described above, it is plausible that previous ChAP-chip analysis determined the genome-wide binding sites of hetero-oligomers consisting of Pmr, TurA, and TurB. Hence, it remains unknown whether the first binding sites of the three MvaT-like proteins are identical. To solve this problem, it is necessary to assess the DNA-binding specificities of the C-terminal domains of Pmr, TurA, and TurB. When these studies are combined with our present results showing the importance of the composition of the hetero-oligomers, they will provide more detail regarding molecular mechanisms underlying the functions of the three MvaT-like proteins. Such information would help to explain the biological meaning of the existence of multiple H-NS family proteins in a cell.

## Materials and Methods

### Bacterial strains, plasmids, and growth conditions

*E. coli* DH5α (Toyobo, Osaka, Japan) and *E. coli* BL21(DE3) (Novagen, San Diego, CA, USA) were used as hosts for plasmid construction and protein expression, respectively. *E. coli* cells harboring the appropriate plasmid were grown in Luria broth (LB) supplemented with 50 µg/ml kanamycin (Km). The properties of plasmid vectors used in the present study are summarized in Table S1.

### Plasmid construction

Construction of the expression plasmids for Pmr, Pmr_ct_46_ (previously named as Pmr_ct), TurA, and TurB (pET-C-His-pmr, pET-N-His-pmr_ct_46_, pET-C-His-turA, and pET-C-His-turB, respectively) was described previously [27], [29]. The other plasmids used in the current study were constructed as follows.

#### pET-C-His-pmr_nt55, pET-C-His-pmr_nt61, and pET-C-His-pmr_nt73

To construct the plasmids for the expression of Pmr_nt_55_, Pmr_nt_61_ and Pmr_nt_73_ with the C-terminal 6× His-tag, the inserts were amplified by PCR using the primers listed in Table S2 with pET-C-His-pmr as the template, and the inserts were ligated into the NdeI and XhoI sites of the pET-26b(+) (Novagen) vector.

#### pET-C-His-pmr_ct58

Construction of the plasmid for the expression of Pmr_ct_58_ with the C-terminal 6× His-tag was performed using the KOD -Plus- Mutagenesis Kit (Toyobo) following the manufacturer’s recommendations. Inverse PCR was performed using the primers listed in Table S2 with pET-C-His-pmr as the template.

#### Derivatives of pET-C-His-pmr, pET-C-His-pmr_nt61, and pET-C-His-pmr_nt73

Site-directed mutagenesis of the coding region of pET-C-His-pmr, pET-C-His-pmr_nt_61_, and pET-C-His-pmr_nt_73_ was performed using the primers listed in Table S2 and the QuikChange Site-directed Mutagenesis Kit (Stratagene, Santa Clara, CA, USA) following the manufacturer’s recommendations.

### Protein expression and purification

The method for expression and purification of Pmr, Pmr_ct_46_, TurA, and TurB was described previously [27], [29]. For the other proteins, transformed *E. coli* BL21(DE3) harboring the various expression plasmids were grown at 25 or 30°C to a turbidity (at 600 nm) of 0.6–0.8, and then heterologous expression of the proteins was induced by the addition of isopropyl β-d-thiogalactopyranoside (IPTG), followed by further overnight cultivation. All purification procedures were carried out at 4°C. The temperature, final concentration of IPTG, and method of purification employed were as follows.

#### Pmr_nt55

Cells were grown at 30°C, and IPTG was added to a final concentration of 0.1 mM. Purification was performed with a HiTrap Chelating HP column (GE Healthcare, Buckinghamshire, UK) using an ÄKTA FPLC instrument (GE Healthcare), following the method described previously [29]. Pmr_nt_55_ was eluted at 475 mM imidazole.

#### Pmr_nt61, Pmr_nt73, Pmr_ct58, and their variants except for Pmr_nt61-R20A

Cells were grown at 30°C, and IPTG was added to a final concentration of 0.1 mM. The crude extract, which was prepared by sonication and subsequent centrifugation, was purified using a MagneHis Protein Purification System (Promega, Madison, WI, USA) following the manufacturer’s recommendations.

#### Pmr_nt61-R20A

Cells were grown at 25°C, and IPTG was added to a final concentration of 0.5 mM. Because of the low solubility of Pmr_nt_61_-R20A, expression of this variant was performed on a large scale, and purification was performed using a HiTrap Chelating HP column as described above. Pmr_nt_61_-R20A was eluted at 475 mM imidazole.

#### Pmr-R8A

Expression and purification using a HiTrap Chelating HP column were performed as for Pmr. Thereafter, purification by gel-filtration chromatography was performed according to the method described in the “Gel-filtration chromatography” section.

#### Purification of Pmr as an analyte for SPR studies

Expression and purification were performed as described above with modification of the buffer used in the purification. Buffer A′ (20 mM Tris-HCl (pH 7.5, 4°C), 0.5 M NaCl, 5% glycerol) and buffer B′ (20 mM Tris-HCl (pH 7.5, 4°C), 0.5 M NaCl, 5% glycerol, and 0.5 M imidazole) were used for purification, and Pmr was eluted at 475 mM imidazole.

### Gel-filtration chromatography

Protein solutions after purification using HiTrap Chelating HP column were applied to a HiLoad 16/60 Superdex 200 prep-grade column (GE Healthcare). Prior to loading of the protein sample, the column was equilibrated with buffer B (20 mM Tris-HCl (pH 7.5, 4°C), 0.5 M NaCl, 10% glycerol, and 0.5 M imidazole). For Western blotting, protein samples were separated by Tricine-SDS-PAGE [42] and transferred to a Sequi-Blot PVDF membrane (Bio-Rad, Hercules, CA, USA) by a semi-dry method. Proteins were detected as described previously [27] using Anti-His Antibody (GE Healthcare) as the primary antibody.

### Protein–protein chemical cross-linking

Protein–protein chemical cross-linking with DMS (Sigma-Aldrich, St. Louis, MO, USA) was performed as described previously [29]. All samples were added to the cross-linking buffer at a final concentration of 0.14 mg/ml. Protein samples were resolved by 14% or 16% Tricine-SDS-PAGE [42]. Quantification of the data was performed using the ImageJ software (http://rsb.info.nih.gov/ij/).

### Modeling of the three-dimensional structure of the Pmr_nt_61_ dimer

The structure of a protein whose amino acid sequence shows marked homology (>25% identity) with that of Pmr_nt_61_ has not yet been determined. We queried the fold-recognition servers FUGUE [43] and pGenTHREADER [44], but were unable to identify appropriate template structures for homology modeling. The structures of the N-terminal regions of three H-NS-like proteins, H-NS of *S. enterica* Typhimurium (PDB ID: 1LR1) [35], H-NS of *E. coli* (PDB ID: 1NI8) [45], and VicH of *V. cholerae* (PDB ID: 1OV9) [31], were available, although these proteins were not included in the results of the above servers due to the low sequence similarity to Pmr_nt_61_ (see Figure S2A). However, Pmr_nt_61_ shares the following characteristics with these proteins. According to the secondary structure prediction by the Jpred server [46], the structure of Pmr_nt_61_ is alpha-helical, similar to the structures of the three H-NS-like proteins. These proteins exist as dimers and are involved in oligomerization. Hence, the structure of Pmr_nt_61_ can be assumed to be similar to those of the H-NS-like proteins. Here, we constructed the structural model of Pmr_nt_61_ based on the crystal structure of the N-terminal region of VicH (VicH_Nt). Automatic sequence alignment programs could not generate appropriate sequence alignments, as shown in Figure S2A, where there are large gaps in the N-terminal region of the Pmr sequence. Therefore, we manually aligned the amino acid sequence of Pmr_nt_61_ to that of VicH_Nt so that L28, L32, L35, and V39 of VicH, which are conserved in the H-NS-like proteins and are important for homodimerization [8], correspond to hydrophobic residues of Pmr_nt_61_ (Figure S2B). Next, we generated structural models using Modeller 9v7 [47], optimized the side-chain conformations using SCWRL 3 [48], and evaluated the models using Verify 3D [49], [50]. The model with the best Verify 3D score was used as the structural model of Pmr_nt_61_.

### Biotinylation of Pmr, TurA, and TurB

Biotinylation of the primary amines in Pmr, TurA, and TurB was carried out using EZ-Link Sulfo-NHS-LC-Biotin (Thermo Scientific, Waltham, MA, USA). 200 µl of purified Pmr, TurA, and TurB solution were dissolved in 800 µl of 10× phosphate-buffered saline containing EZ-Link Sulfo-NHS-LC-Biotin (100-fold molar equivalents of Pmr, TurA, and TurB) and incubated at room temperature for 30 min. The biotinylated proteins were purified using the MagneHis Protein Purification System (Promega) using a biotinylation wash buffer (20 mM Tris-HCl (pH 7.5, 4°C), 0.5 M NaCl, 10% glycerol, and 0.1 M imidazole) for washing and buffer B for elution.

### SPR studies

A Biacore 2000 system and the BIACORE 2000 Control Software version 3.2.1 (GE Healthcare) were used for SPR studies. A streptavidin-coated sensor chip SA was used to immobilize the biotinylated samples. A sensor chip was pretreated with three consecutive 10-µl injections of 50 mM NaOH in 1 M NaCl to remove nonspecifically bound contaminants. The injection of biotinylated samples onto the sensor surface was controlled to obtain a response of ∼500 RU at a flow rate of 10 µl/min. We used nearly identical quantities of immobilized proteins in the three flow cells. The measurement was performed at 4°C in running buffer (20 mM Tris-HCl (pH 7.5, 4°C), 0.5 M NaCl, 5% glycerol, and 475 mM imidazole), and the flow rate was maintained at 30 µl/min. The Pmr analyte, which was purified as described above, was allowed to interact with immobilized proteins for 7 min, constituting the association phase. Dissociation of Pmr, constituting the dissociation phase, was allowed to proceed for an additional 3 min, after which the sensor chip was regenerated by injecting regeneration buffer (10 mM glycine-HCl (pH 1.5), 0.5 M NaCl, 10% glycerol, and 0.5 M imidazole). One control surface was routinely treated in the same manner as the immobilized surface, except that the immobilization step was omitted. The sensorgrams were evaluated using BIAevaluation, version 4.1.1 (GE Healthcare). *R*_eq_ was calculated from the sensorgrams, from which the SPR signals were calculated by reference subtraction of the signals from the control surface.

## Supporting Information

Figure S1 **Effects of alanine substitutions on the homo-oligomerization capacity of Pmr_nt_61_.** Tricine-SDS-PAGE profiles of Pmr_nt_61_ and its alanine-substituted variants after chemical cross-linking are shown. The analyses were performed using DMS as a cross-linker. Numbers indicate incubation durations (min), and “M” indicates the protein marker. The seven oligomerization-deficient variants are shown in red.
(PDF)

Figure S2 **Alignments of amino acid sequences of H-NS-like proteins and Pmr.** (A) Amino acid sequences of H-NS of *S. enterica* Typhimurium, VicH of *V. cholerae*, and Pmr that were aligned automatically using the CLUSTAL W software, version 2.1. The amino acid residues identical in two or all three members are shown in red. The dimerization/oligomerization domain and DNA-binding domain of Pmr are highlighted in light blue and pink, respectively. The seven residues important for homo-oligomerization of Pmr in this study are highlighted in orange. Secondary structural features of the N-terminal region of H-NS determined by Arold *et al.* [32] are shown: gray line, unstructured loop; green box, α helix; H1–H4, names of the helices. (B) Manual alignment of amino acid sequences of Pmr_nt_61_ and VicH_Nt (residues 4–53). L28, L32, L35, and V39 of VicH_Nt were manually aligned to F36, L40, L43, and Y47 of Pmr_nt_61_, which are shown in green in the panel.
(TIF)

Figure S3 **The binding amount of ^A^Pmr kept increasing when higher ^A^Pmr concentrations were used.** The figure presents the binding amount of ^A^Pmr per one homodimer of ^L^Pmr (pink), ^L^TurA (blue), and ^L^TurB (green) as a function of the concentration of ^A^Pmr (µM). The amount of ^L^Pmr, ^L^TurA, and ^L^TurB, which were immobilized on a sensor chip, are indicated in each panel. To calculate the binding amount of ^A^Pmr per one homodimer of ligands, the equilibrium binding response (*R*_eq_) was divided by the amount of immobilized ^L^Pmr, ^L^TurA, and ^L^TurB. Three representative data from independent experiments are shown (A–C).
(TIF)

Table S1 **Plasmid vectors used in this study.**
(PDF)

Table S2 **Oligonucleotide primers used for genetic constructions.**
(PDF)
