# Supplementary material for: Oligomerization Mechanisms of an H-NS Family Protein, Pmr, Encoded on the Plasmid pCAR1 Provide a Molecular Basis for Functions of H-NS Family Members
Source: PLoS One. 2014 Aug 19;9(8):e105656. doi: 10.1371/journal.pone.0105656 (PMC4138198; doi:10.1371/journal.pone.0105656)
Supplement: Figure S1 — Effects of alanine substitutions on the homo-oligomerization capacity of Pmr_nt61. Tricine-SDS-PAGE profiles of Pmr_nt61 and its alanine-substituted variants after chemical cross-linking are shown. The analyses were performed using DMS as a cross-linker. Numbers indicate incubation durations (min), and “M” indicates the protein marker. The seven oligomerization-deficient variants are shown in red. (PDF) [file pone.0105656.s001.pdf]

Figure S1

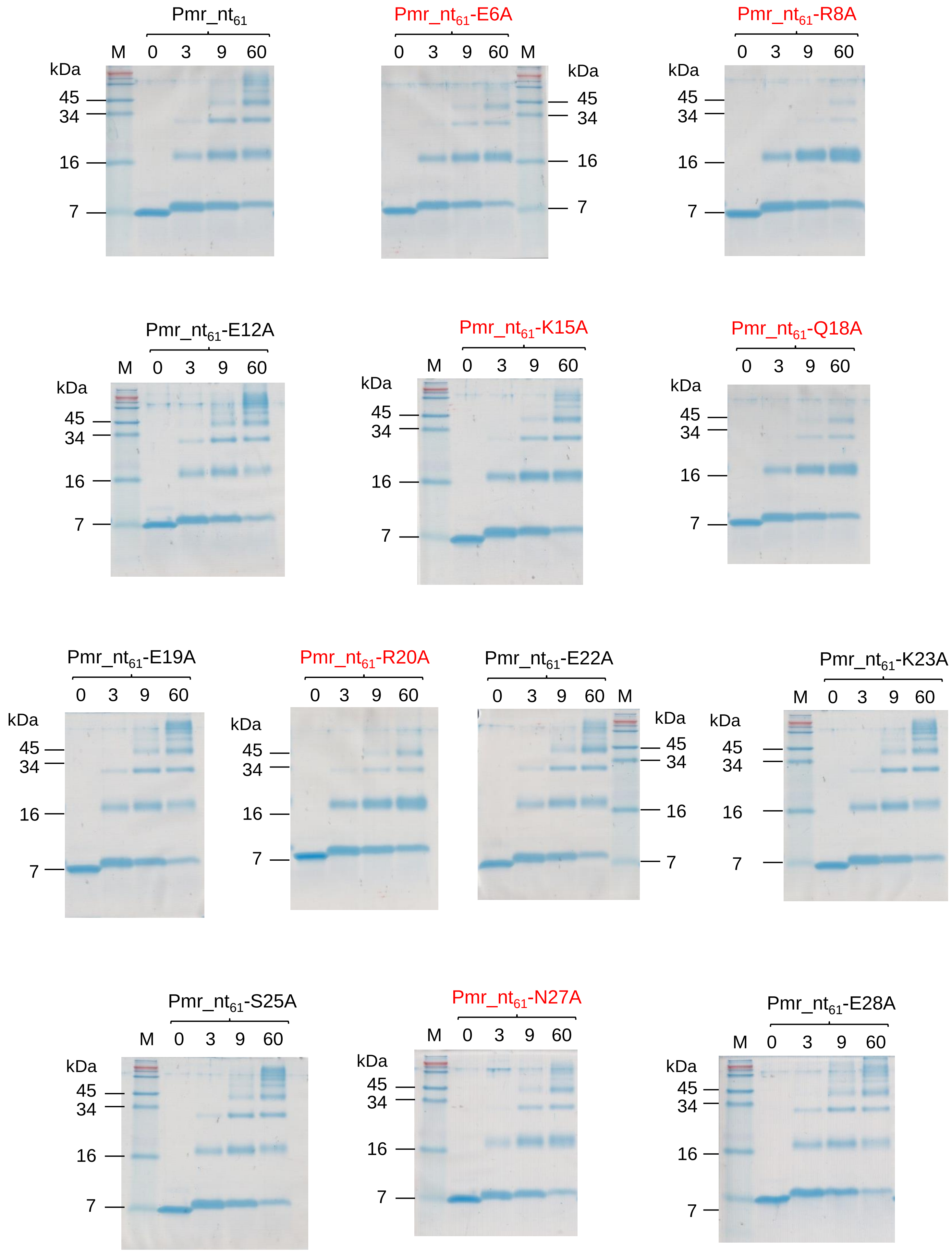

Pmr_nt$_{61}$
M 0 3 9 60
kDa
45
34
16
7
Pmr_nt$_{61}$-E6A
0 3 9 60 M
kDa
45
34
16
7
Pmr_nt$_{61}$-R8A
0 3 9 60
kDa
45
34
16
7
Pmr_nt$_{61}$-E12A
M 0 3 9 60
kDa
45
34
16
7
Pmr_nt$_{61}$-K15A
M 0 3 9 60
kDa
45
34
16
7
Pmr_nt$_{61}$-Q18A
0 3 9 60
kDa
45
34
16
7
Pmr_nt$_{61}$-E19A
0 3 9 60
kDa
45
34
16
7
Pmr_nt$_{61}$-R20A
0 3 9 60
kDa
45
34
16
7
Pmr_nt$_{61}$-E22A
0 3 9 60 M
kDa
45
34
16
7
Pmr_nt$_{61}$-K23A
M 0 3 9 60
kDa
45
34
16
7
Pmr_nt$_{61}$-S25A
M 0 3 9 60
kDa
45
34
16
7
Pmr_nt$_{61}$-N27A
M 0 3 9 60
kDa
45
34
16
7
Pmr_nt$_{61}$-E28A
M 0 3 9 60
kDa
45
34
16
7

Figure S1 (continued)

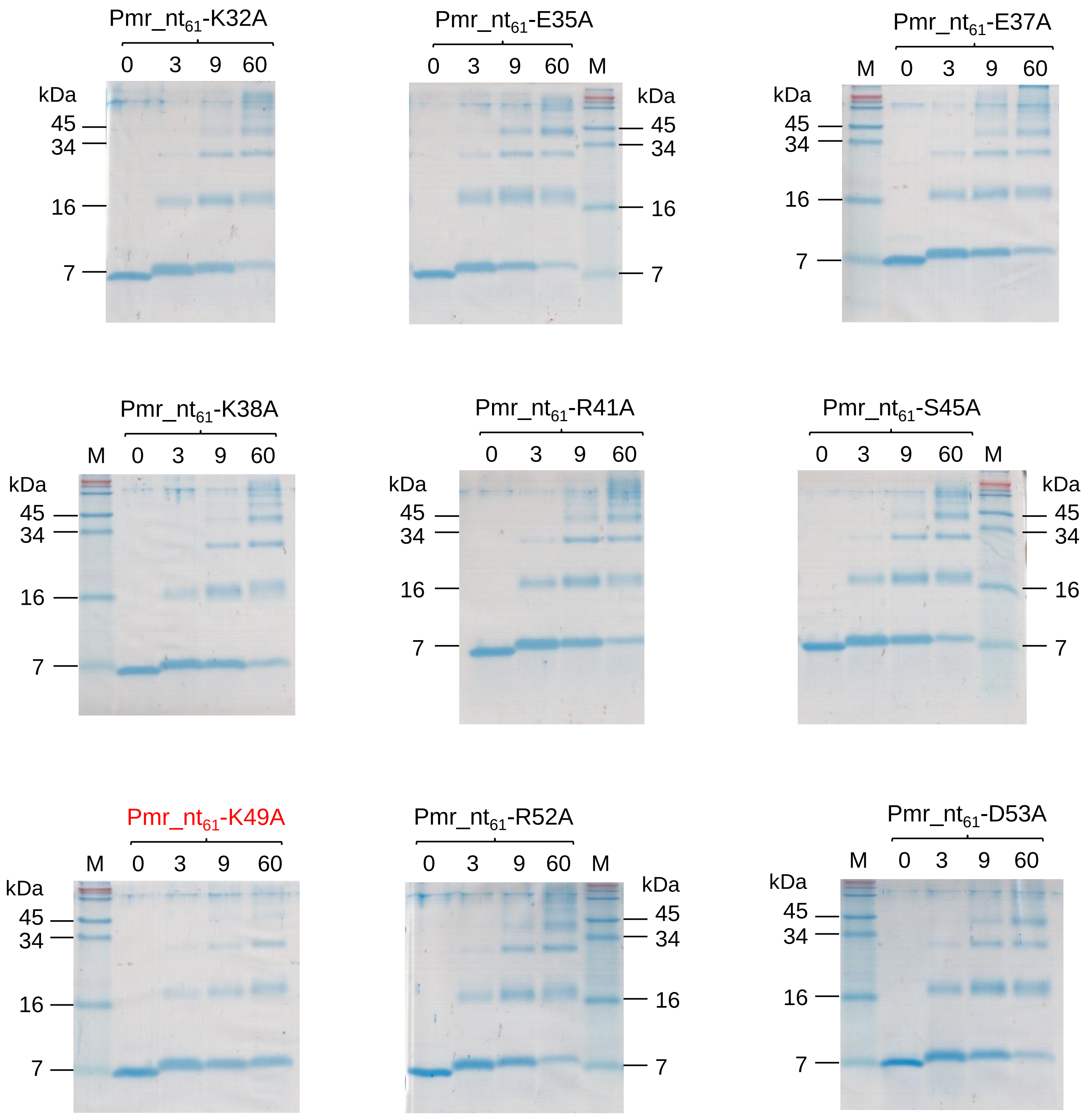

Figure S1. Effects of alanine substitutions on the homo-oligomerization capacity of Pmr_nt$_{61}$. Tricine-SDS-PAGE profiles of Pmr_nt$_{61}$ and its alanine-substituted variants after chemical cross-linking are shown. The analyses were performed using DMS as a cross-linker. Numbers indicate incubation durations (min), and “M” indicates the protein marker. The seven oligomerization-deficient variants are shown in red.
