# Supplementary figures and images for: Oligomerization Mechanisms of an H-NS Family Protein, Pmr, Encoded on the Plasmid pCAR1 Provide a Molecular Basis for Functions of H-NS Family Members
Source: PLoS One. 2014 Aug 19;9(8):e105656. doi: 10.1371/journal.pone.0105656 (PMC4138198; doi:10.1371/journal.pone.0105656)

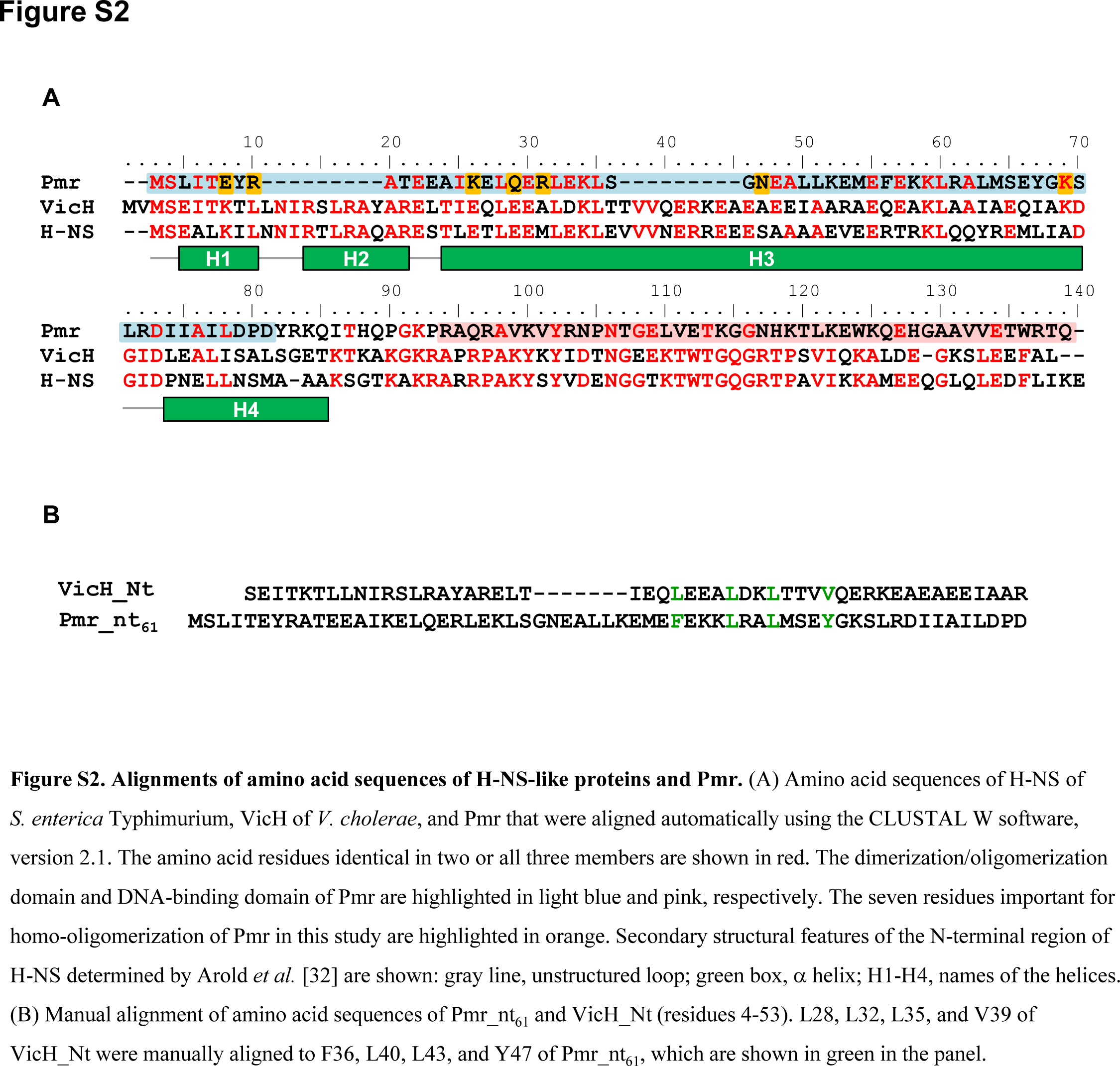

Supplement: Figure S2 — Alignments of amino acid sequences of H-NS-like proteins and Pmr. (A) Amino acid sequences of H-NS of S. enterica Typhimurium, VicH of V. cholerae, and Pmr that were aligned automatically using the CLUSTAL W software, version 2.1. The amino acid residues identical in two or all three members are shown in red. The dimerization/oligomerization domain and DNA-binding domain of Pmr are highlighted in light blue and pink, respectively. The seven residues important for homo-oligomerization of Pmr in this study are highlighted in orange. Secondary structural features of the N-terminal region of H-NS determined by Arold et al. [32] are shown: gray line, unstructured loop; green box, α helix; H1–H4, names of the helices. (B) Manual alignment of amino acid sequences of Pmr_nt61 and VicH_Nt (residues 4–53). L28, L32, L35, and V39 of VicH_Nt were manually aligned to F36, L40, L43, and Y47 of Pmr_nt61, which are shown in green in the panel. (TIF) [file pone.0105656.s002.tif]

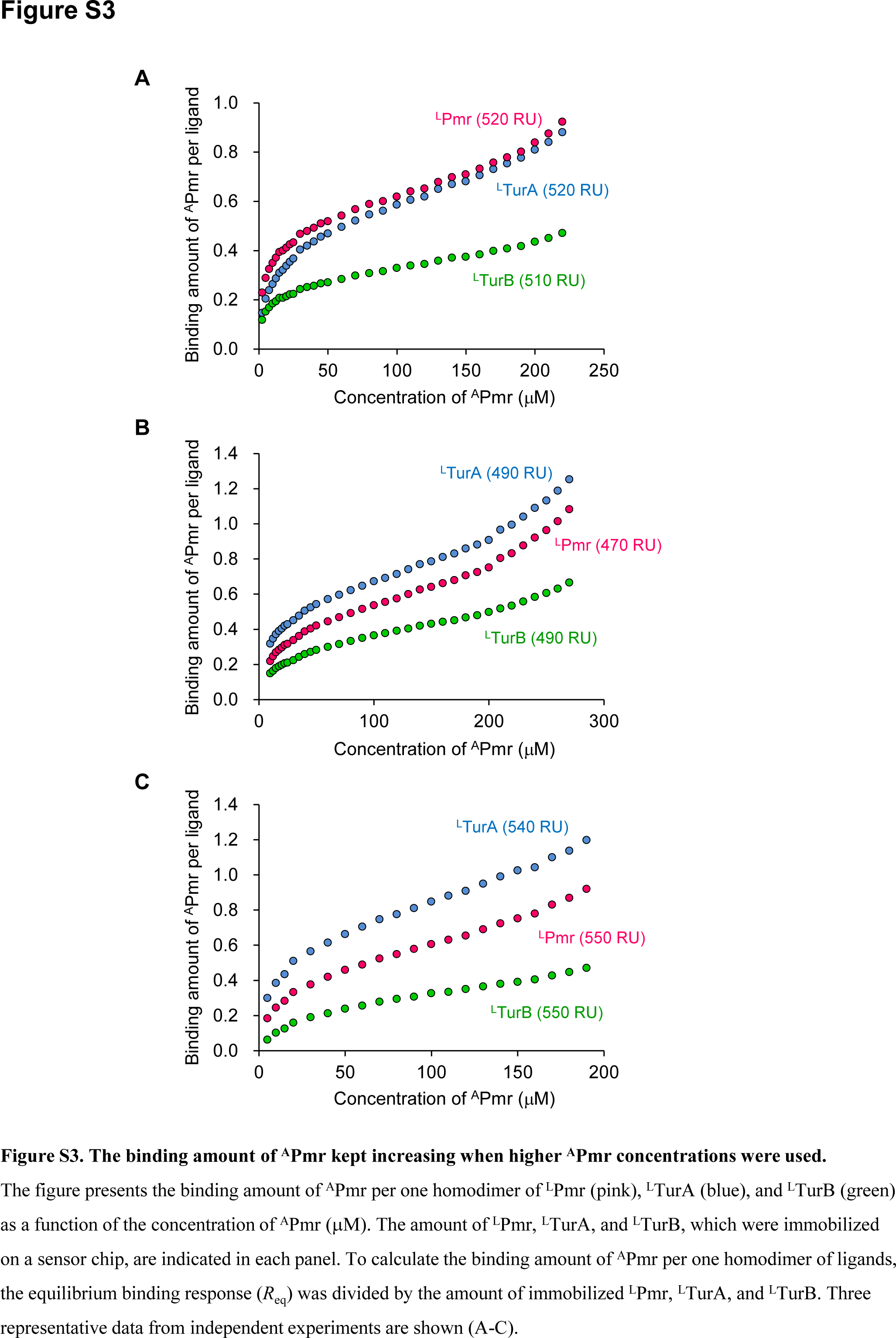

Supplement: Figure S3 — The binding amount of APmr kept increasing when higher APmr concentrations were used. The figure presents the binding amount of APmr per one homodimer of LPmr (pink), LTurA (blue), and LTurB (green) as a function of the concentration of APmr (µM). The amount of LPmr, LTurA, and LTurB, which were immobilized on a sensor chip, are indicated in each panel. To calculate the binding amount of APmr per one homodimer of ligands, the equilibrium binding response (R eq) was divided by the amount of immobilized LPmr, LTurA, and LTurB. Three representative data from independent experiments are shown (A–C). (TIF) [file pone.0105656.s003.tif]
