# Supplementary material for: Oligomerization Mechanisms of an H-NS Family Protein, Pmr, Encoded on the Plasmid pCAR1 Provide a Molecular Basis for Functions of H-NS Family Members
Source: PLoS One. 2014 Aug 19;9(8):e105656. doi: 10.1371/journal.pone.0105656 (PMC4138198; doi:10.1371/journal.pone.0105656)
Supplement: Table S1 — Plasmid vectors used in this study. (PDF) [file pone.0105656.s004.pdf]

Table S1. Plasmid vectors used in this study.

Plasmid vectors	Relevant properties	Reference or Source
pET26b(+)	pBR322 replicon, Km^r, T7 promoter, *lacI*	Novagen
pET-C-His-pmr	pET-26b(+), NdeI-XhoI fragment containing *pmr*	[27]
pET-C-His-turA	pET-26b(+), NdeI-XhoI fragment containing *turA*	[29]
pET-C-His-turB	pET-26b(+), NdeI-XhoI fragment containing *turB*	[29]
pET-C-His-pmr_nt_{55}	pET26b(+), NdeI-XhoI fragment containing the gene encoding the N-terminal part of Pmr (residues 1-55)	This study
pET-C-His-pmr_nt_{61}	pET26b(+), NdeI-XhoI fragment containing the gene encoding the N-terminal part of Pmr (residues 1-61)	This study
pET-C-His-pmr_nt_{73}	pET26b(+), NdeI-XhoI fragment containing the gene encoding the N-terminal part of Pmr (residues 1-73)	This study
pET-C-His-pmr_ct_{58}	pET26b(+), NdeI-XhoI fragment containing the gene encoding the C-terminal part of Pmr (residues 62-119)	This study
pET-N-His-pmr_ct_{46}	pET26b(+), NdeI-XhoI fragment containing the gene encoding the C-terminal part of Pmr (residues 74-119) with 6 × His at its N-terminus	[29]
pET-C-His-pmr_nt_{61}-E6A	pET-C-His-pmr_nt_{61} derivative, triplet codon for E6 is replaced with GCG	This study
pET-C-His-pmr_nt_{61}-R8A	pET-C-His-pmr_nt_{61} derivative, triplet codon for R8 is replaced with GCG	This study
pET-C-His-pmr_nt_{61}-E12A	pET-C-His-pmr_nt_{61} derivative, triplet codon for E12 is replaced with GCG	This study
pET-C-His-pmr_nt_{61}-K15A	pET-C-His-pmr_nt_{61} derivative, triplet codon for K15 is replaced with GCG	This study
pET-C-His-pmr_nt_{61}-E16A	pET-C-His-pmr_nt_{61} derivative, triplet codon for E16 is replaced with GCG	This study
pET-C-His-pmr_nt_{61}-Q18A	pET-C-His-pmr_nt_{61} derivative, triplet codon for Q18 is replaced with GCG	This study
pET-C-His-pmr_nt_{61}-E19A	pET-C-His-pmr_nt_{61} derivative, triplet codon for E19 is replaced with GCG	This study
pET-C-His-pmr_nt_{61}-R20A	pET-C-His-pmr_nt_{61} derivative, triplet codon for R20 is replaced with GCG	This study

Table S1. (continued)

Plasmid vectors	Relevant properties	Reference or Source
pET-C-His-pmr_nt$_{61}$-E22A	pET-C-His-pmr_nt$_{61}$ derivative, triplet codon for E22 is replaced with GCG	This study
pET-C-His-pmr_nt$_{61}$-K23A	pET-C-His-pmr_nt$_{61}$ derivative, triplet codon for K23 is replaced with GCG	This study
pET-C-His-pmr_nt$_{61}$-S25A	pET-C-His-pmr_nt$_{61}$ derivative, triplet codon for S25 is replaced with GCG	This study
pET-C-His-pmr_nt$_{61}$-N27A	pET-C-His-pmr_nt$_{61}$ derivative, triplet codon for N27 is replaced with GCG	This study
pET-C-His-pmr_nt$_{61}$-E28A	pET-C-His-pmr_nt$_{61}$ derivative, triplet codon for E28 is replaced with GCG	This study
pET-C-His-pmr_nt$_{61}$-K32A	pET-C-His-pmr_nt$_{61}$ derivative, triplet codon for K32 is replaced with GCG	This study
pET-C-His-pmr_nt$_{61}$-E35A	pET-C-His-pmr_nt$_{61}$ derivative, triplet codon for E35 is replaced with GCG	This study
pET-C-His-pmr_nt$_{61}$-E37A	pET-C-His-pmr_nt$_{61}$ derivative, triplet codon for E37 is replaced with GCG	This study
pET-C-His-pmr_nt$_{61}$-K38A	pET-C-His-pmr_nt$_{61}$ derivative, triplet codon for K38 is replaced with GCG	This study
pET-C-His-pmr_nt$_{61}$-R41A	pET-C-His-pmr_nt$_{61}$ derivative, triplet codon for R41 is replaced with GCG	This study
pET-C-His-pmr_nt$_{61}$-S45A	pET-C-His-pmr_nt$_{61}$ derivative, triplet codon for S45 is replaced with GCG	This study
pET-C-His-pmr_nt$_{61}$-K49A	pET-C-His-pmr_nt$_{61}$ derivative, triplet codon for K49 is replaced with GCG	This study
pET-C-His-pmr_nt$_{61}$-R52A	pET-C-His-pmr_nt$_{61}$ derivative, triplet codon for R52 is replaced with GCG	This study
pET-C-His-pmr_nt$_{61}$-D53A	pET-C-His-pmr_nt$_{61}$ derivative, triplet codon for D53 is replaced with GCG	This study
pET-C-His-pmr_nt$_{73}$-R8A	pET-C-His-pmr_nt$_{73}$ derivative, triplet codon for R8 is replaced with GCG	This study
pET-C-His-pmr-R8A	pET-C-His-pmr derivative, triplet codon for R8 is replaced with GCG	This study
