# Supplementary material for: Oligomerization Mechanisms of an H-NS Family Protein, Pmr, Encoded on the Plasmid pCAR1 Provide a Molecular Basis for Functions of H-NS Family Members
Source: PLoS One. 2014 Aug 19;9(8):e105656. doi: 10.1371/journal.pone.0105656 (PMC4138198; doi:10.1371/journal.pone.0105656)
Supplement: Table S2 — Oligonucleotide primers used for genetic constructions. (PDF) [file pone.0105656.s005.pdf]

Table S2. Oligonucleotide primers used for genetic constructions.

Primer	Sequence (5’ → 3’)[a]	Plasmid vectors constructed using the primer
C-FLAG-Pmr-F [27]	CATATGTCCCTCATCACCGA	pET-C-His-pmr_nt$_{55}$,<br>pET-C-His-pmr_nt$_{61}$,<br>pET-C-His-pmr_nt$_{73}$
Pmr_nt$_{55}$ C-His-R	CTCGAGGATGATGTCGCGCAGGC	pET-C-His-pmr_nt$_{55}$
Pmr_nt$_{61}$ C-His-R	CTCGAGGTCCGGATCAAGAATC	pET-C-His-pmr_nt$_{61}$
Pmr_nt$_{73}$ C-His-R	CTCGAGAGGTTTGCCAGGCTGAT	pET-C-His-pmr_nt$_{73}$
Pmr_ct$_{58}$ C-His-F	TATCGAAAGCAAATTACCCA	pET-C-His-pmr_ct$_{58}$
Pmr_ct$_{58}$ C-His-R	CATATGTATATCTCCTTCTT	pET-C-His-pmr_ct$_{58}$
Pmr E6A-F	GTCCCTCATCACCG**CG**TACCGAGCCACAG	pET-C-His-pmr_nt$_{61}$-E6A
Pmr E6A-R	CTGTGGCTCGGTA**CG**CGGTGATGAGGGAC	pET-C-His-pmr_nt$_{61}$-E6A
Pmr R8A-F	CATCACCGAATAC**GCG**GCCACAGAGGAAGC	pET-C-His-pmr_nt$_{61}$-R8A,<br>pET-C-His-pmr_nt$_{73}$-R8A,<br>pET-C-His-pmr-R8A,
Pmr R8A-R	GCTTCCTCTGTGGC**CGC**GTATTCGGTGATG	pET-C-His-pmr_nt$_{61}$-R8A,<br>pET-C-His-pmr_nt$_{73}$-R8A,<br>pET-C-His-pmr-R8A,
Pmr E12A-F	CCGAGCCACAGAGG**CG**GCCATCAAGGAGC	pET-C-His-pmr_nt$_{61}$-E12A
Pmr E12A-R	GCTCCTTGATGGC**CG**CCTCTGTGGCTCGG	pET-C-His-pmr_nt$_{61}$-E12A
Pmr K15A-F	GAGGAAGCCATC**GC**GGAGCTTCAGGAACG	pET-C-His-pmr_nt$_{61}$-K15A
Pmr K15A-R	CGTTCCTGAAGCTCC**GC**GATGGCTTCCTC	pET-C-His-pmr_nt$_{61}$-K15A
Pmr E16A-F	GGAAGCCATCAAGG**C**GCTTCAGGAACGAC	pET-C-His-pmr_nt$_{61}$-E16A
Pmr E16A-R	GTCGTTCCTGAAGC**G**CCTTGATGGCTTCC	pET-C-His-pmr_nt$_{61}$-E16A
Pmr Q18A-F	CCATCAAGGAGCTT**GC**GGAACGACTGGAG	pET-C-His-pmr_nt$_{61}$-Q18A
Pmr Q18A-R	CTCCAGTCGTTCC**GC**AAGCTCCTTGATGG	pET-C-His-pmr_nt$_{61}$-Q18A
Pmr E19A-F	CAAGGAGCTTCAGG**CG**CGACTGGAGAAGC	pET-C-His-pmr_nt$_{61}$-E19A
Pmr E19A-R	GCTTCTCCAGTCG**CG**CCTGAAGCTCCTTG	pET-C-His-pmr_nt$_{61}$-E19A
Pmr R20A-F	GGAGCTTCAGGAA**GCG**CTGGAGAAGCTGAG	pET-C-His-pmr_nt$_{61}$-R20A
Pmr R20A-R	CTCAGCTTCTCCAG**CGC**TTCCTGAAGCTCC	pET-C-His-pmr_nt$_{61}$-R20A
Pmr E22A-F	CAGGAACGACTGG**C**GAAGCTGAGCGG	pET-C-His-pmr_nt$_{61}$-E22A
Pmr E22A-R	CCGCTCAGCTTC**G**CCAGTCGTTCCTG	pET-C-His-pmr_nt$_{61}$-E22A
Pmr K23A-F	GGAACGACTGGAG**GC**GCTGAGCGGAAATG	pET-C-His-pmr_nt$_{61}$-K23A
Pmr K23A-R	CATTTCCGCTCAGC**GC**CTCCAGTCGTTCC	pET-C-His-pmr_nt$_{61}$-K23A
Pmr S25A-F	GACTGGAGAAGCTG**GCG**GGAAATGAAGCCC	pET-C-His-pmr_nt$_{61}$-S25A

Table S2. (continued)

Primer	Sequence (5’ → 3’)[a]	Plasmid vectors constructed using the primer
Pmr S25A-R	GGGCTTCATTTCC**CGC**CAGCTTCTCCAGTC	pET-C-His-pmr_nt$_{61}$-S25A
Pmr N27A-F	GAGAAGCTGAGCGGA**GCG**GAAGCCCTTTTG	pET-C-His-pmr_nt$_{61}$-N27A
Pmr N27A-R	CAAAAGGGCTTC**CGC**TCCGCTCAGCTTCTC	pET-C-His-pmr_nt$_{61}$-N27A
Pmr E28A-F	CTGAGCGGAAATG**CG**GCCCTTTTGAAAGAG	pET-C-His-pmr_nt$_{61}$-E28A
Pmr E28A-R	CTCTTTCAAAAGGGC**CG**CATTTCCGCTCAG	pET-C-His-pmr_nt$_{61}$-E28A
Pmr K32A-F	GAAGCCCTTTTG**GCG**GAGATGGAGTTCGAG	pET-C-His-pmr_nt$_{61}$-K32A
Pmr K32A-R	CTCGAACTCCATCTC**CGC**CAAAAGGGCTTC	pET-C-His-pmr_nt$_{61}$-K32A
Pmr E35A-F	CTTTTGAAAGAGATGG**C**GTTCGAGAAAAAG	pET-C-His-pmr_nt$_{61}$-E35A
Pmr E35A-R	CTTTTTCTCGAAC**G**CCATCTCTTTCAAAAG	pET-C-His-pmr_nt$_{61}$-E35A
Pmr E37A-F	GAGATGGAGTTCG**C**GAAAAAGCTGCGGGCC	pET-C-His-pmr_nt$_{61}$-E37A
Pmr E37A-R	GGCCCGCAGCTTTTTC**G**CGAACTCCATCTC	pET-C-His-pmr_nt$_{61}$-E37A
Pmr K38A-F	GAGATGGAGTTCGAG**GCG**AAGCTGCGGGCC	pET-C-His-pmr_nt$_{61}$-K38A
Pmr K38A-R	GGCCCGCAGCTT**CGC**CTCGAACTCCATCTC	pET-C-His-pmr_nt$_{61}$-K38A
Pmr R41A-F	GAGAAAAAGCTG**GC**GGCCTTGATGTCTGAG	pET-C-His-pmr_nt$_{61}$-R41A
Pmr R41A-R	CTCAGACATCAAGGCC**GC**CAGCTTTTTCTC	pET-C-His-pmr_nt$_{61}$-R41A
Pmr S45A-F	CGGGCCTTGATG**G**C**G**GAGTATGGCAAGAGC	pET-C-His-pmr_nt$_{61}$-S45A
Pmr S45A-R	GCTCTTGCCATACTC**C**G**C**CATCAAGGCCCG	pET-C-His-pmr_nt$_{61}$-S45A
Pmr K49A-F	GTCTGAGTATGGC**GC**GAGCCTGCGCGACATC	pET-C-His-pmr_nt$_{61}$-K49A
Pmr K49A-R	GATGTCGCGCAGGCTC**GC**GCCATACTCAGAC	pET-C-His-pmr_nt$_{61}$-K49A
Pmr R52A-F	GTATGGCAAGAGCCTG**GCG**GACATCATCGC	pET-C-His-pmr_nt$_{61}$-R52A
Pmr R52A-R	GCGATGATGTC**CGC**CAGGCTCTTGCCATAC	pET-C-His-pmr_nt$_{61}$-R52A
Pmr D53A-F	GCAAGAGCCTGCGCG**CG**ATCATCGCGATTC	pET-C-His-pmr_nt$_{61}$-D53A
Pmr D53A-R	GAATCGCGATGAT**CG**CGCGCAGGCTCTTGC	pET-C-His-pmr_nt$_{61}$-D53A

[a] NdeI and XhoI restriction sites are underlined; mutations are in bold.
